# Supplementary material for: Modulating mitofusins to control mitochondrial function and signaling
Source: Nat Commun. 2022 Jul 7;13:3775. doi: 10.1038/s41467-022-31324-1 (PMC9262907; doi:10.1038/s41467-022-31324-1)
Supplement: Supplementary file 1 — Supplemmentary Information [file 41467_2022_31324_MOESM1_ESM.pdf]

Supplementary Information

Modulating mitofusins to control mitochondrial function and signaling

Emmanouil Zacharioudakis[1-5], Bogos Agianian[1-5], Vasantha Kumar MV[1-5], Nikolaos Biris[1-5], Thomas P. Garner[1-5], Inna Rabinovich-Nikitin[6-8], Amanda T. Ouchida[1-5] , Victoria Margulets[6-8], Lars Ulrik Nordstrøm[1], Joel S. Riley[9,10], Igor Dolgalev[11-13], Yun Chen[2-4,14,15], Andre J. H. Wittig[2-4,14,15], Ryan Pekson[2-4,14,15], Chris Mathew[1], Peter Wei[1], Aristotelis Tsirigos[11-13], Stephen W.G. Tait[9-10], Lorrie A. Kirshenbaum[6-8], Richard N. Kitsis[2-4,14,15], Evripidis Gavathiotis[1-5*]

[1]Department of Biochemistry, Albert Einstein College of Medicine, Bronx, NY, USA
[2]Department of Medicine, Albert Einstein College of Medicine, Bronx, NY, USA
[3]Wilf Family Cardiovascular Research Institute, Albert Einstein College of Medicine, Bronx, NY, USA
[4]Albert Einstein Cancer Center, Albert Einstein College of Medicine, Bronx, NY, USA
[5]Institute for Aging Research, Albert Einstein College of Medicine, Bronx, NY, USA
[6]Department of Physiology and Pathophysiology, Max Rady College of Medicine, Faculty of Health Sciences, University of Manitoba, Winnipeg, Manitoba, Canada
[7]Department of Pharmacology and Therapeutics, Max Rady College of Medicine, Faculty of Health Sciences, University of Manitoba, Winnipeg, Manitoba, Canada
[8]Institute of Cardiovascular Sciences, St. Boniface Research Centre, Winnipeg, Manitoba, Canada
[9]Cancer Research UK Beatson Institute, Glasgow, UK
[10]Institute of Cancer Sciences, University of Glasgow, Glasgow, UK
[11]Department of Pathology, New York University School of Medicine, New York, NY, USA
[12]Laura & Isaac Perlmutter Cancer Center, New York University School of Medicine, New York, NY, USA
[13]Applied Bioinformatics Laboratories, New York University School of Medicine, New York, NY, USA
[14]Department of Cell Biology, Albert Einstein College of Medicine, Bronx, NY, USA
[15]Einstein-Mount Sinai Diabetes Research Center, Albert Einstein College of Medicine, Bronx, NY, USA

*to whom correspondence should be addressed:

evripidis.gavathiotis@einsteinmed.edu

Contents:

Supplementary Figures 1-16
Supplementary Tables 1-3

a

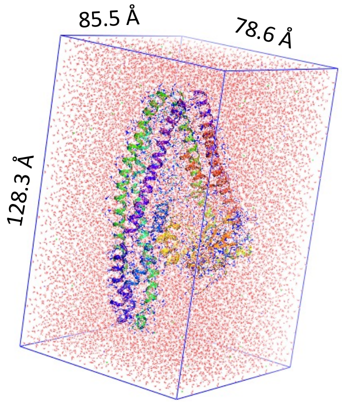

Protein atoms:	12178 (757 residues)
Solvent (water) atoms:	68031
Ions (Na+, Cl-):	173
Total atoms:	80382

b

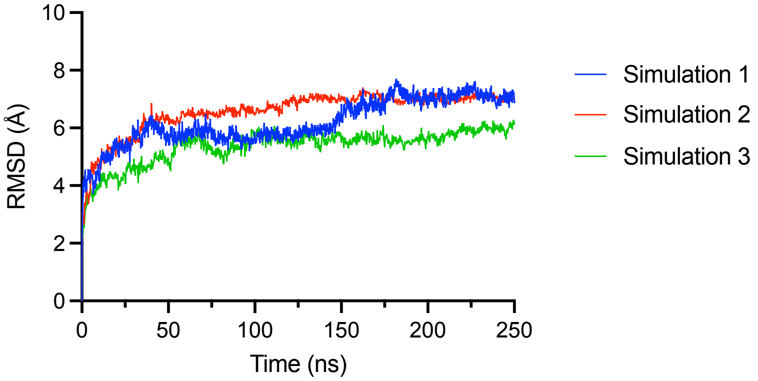

Supplementary Fig. 1. Molecular dynamics simulations of MFN2.

a, Solvated (TIP3P waters) orthorhombic box used in molecular dynamics simulations of MFN2. The full-length MFN2 model is shown in cartoons. The number of atoms used in the simulations is also shown. **b**, RMSD plot of protein Cα atoms from three independent 250 ns molecular dynamics simulations of the MFN2 system indicating convergence. Source data are provided as a Source Data file.

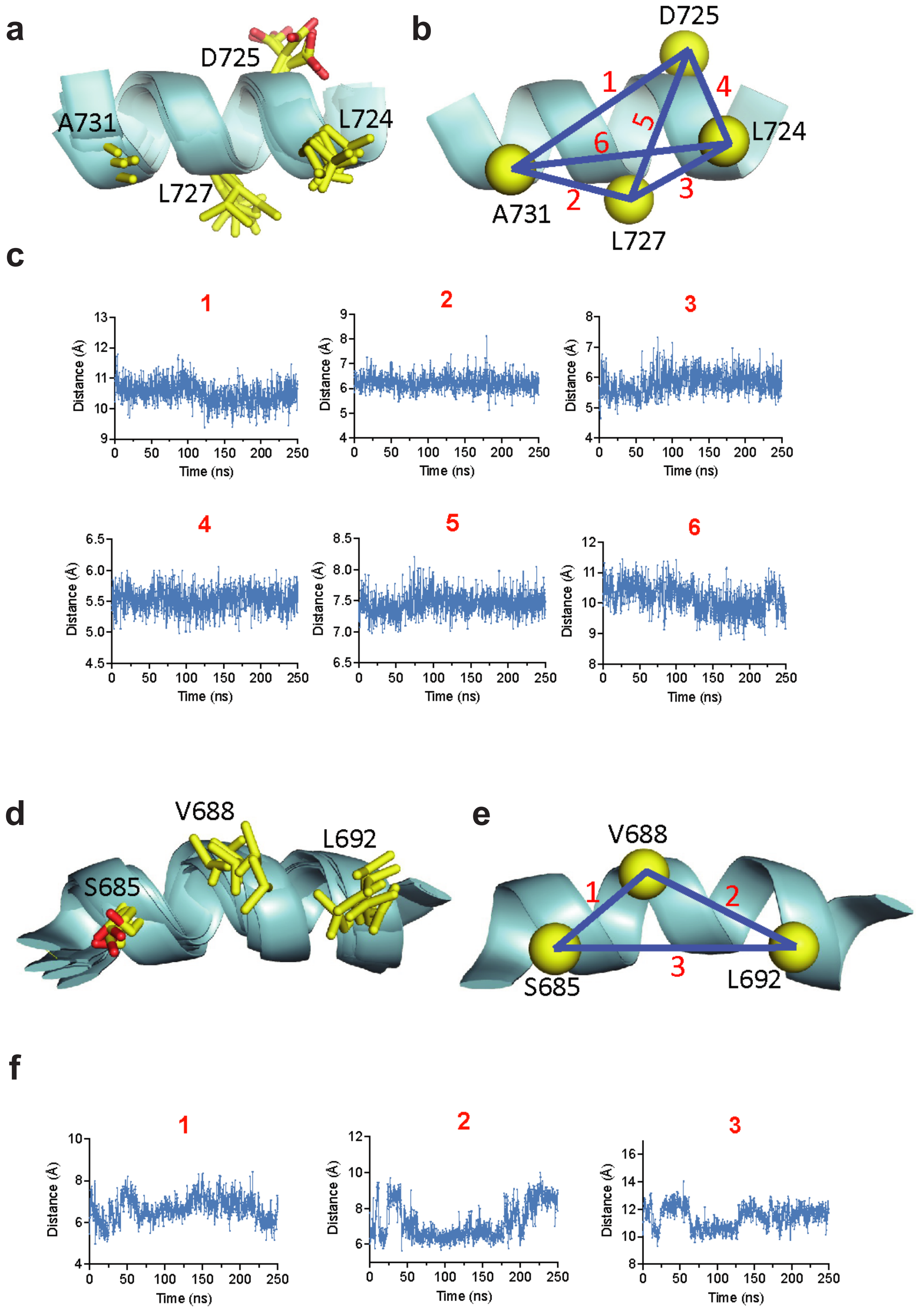
a
D725
A731
L724
L727
b
D725
1
4
5
6
L724
A731
2
3
L727
c
1
2
3
4
5
6
Distance (Å)
Time (ns)
d
V688
L692
S685
e
V688
1
2
3
S685
L692
f
1
2
3
Distance (Å)
Time (ns)

Supplementary Fig. 2. Molecular dynamics simulations show weak fluctuations of the HR2 binding zones used in pharmacophore-based in silico screens.

a, Overlaid snapshots of the HR2 binding zone calculated from molecular dynamics (MD) simulations of full-length MFN2 structural model representing 0, 50, 150, 200 and 250 ns from a representative MD simulation showing conformational dynamics of L724, D725, L727 and A731 binding residues in yellow sticks. **b**, Cβ atoms of L724, D725, L727 and A731 binding residues are shown in spheres. Distances between Cβ atoms are numbered. **c**, Plots of Cβ-Cβ distances shown in b, over the course of the 250 ns MD simulation demonstrating spatial rigidity of the binding site residues. **d,** Overlaid snapshots of the HR2 binding zone calculated from molecular dynamics (MD) simulations of intact MFN2 structural model representing 0, 50, 150, 200 and 250 ns from a representative MD simulation showing conformational dynamics of S685, V688 and L692 binding residues in yellow sticks. **e**, Cβ atoms of S685, V688 and L692 binding residues are shown in spheres. Distances between Cβ atoms are numbered. **f**, Plots of Cβ-Cβ distances shown in e, over the course of the 250 ns MD simulation demonstrating spatial rigidity of the binding site residues. Source data are provided as a Source Data file.

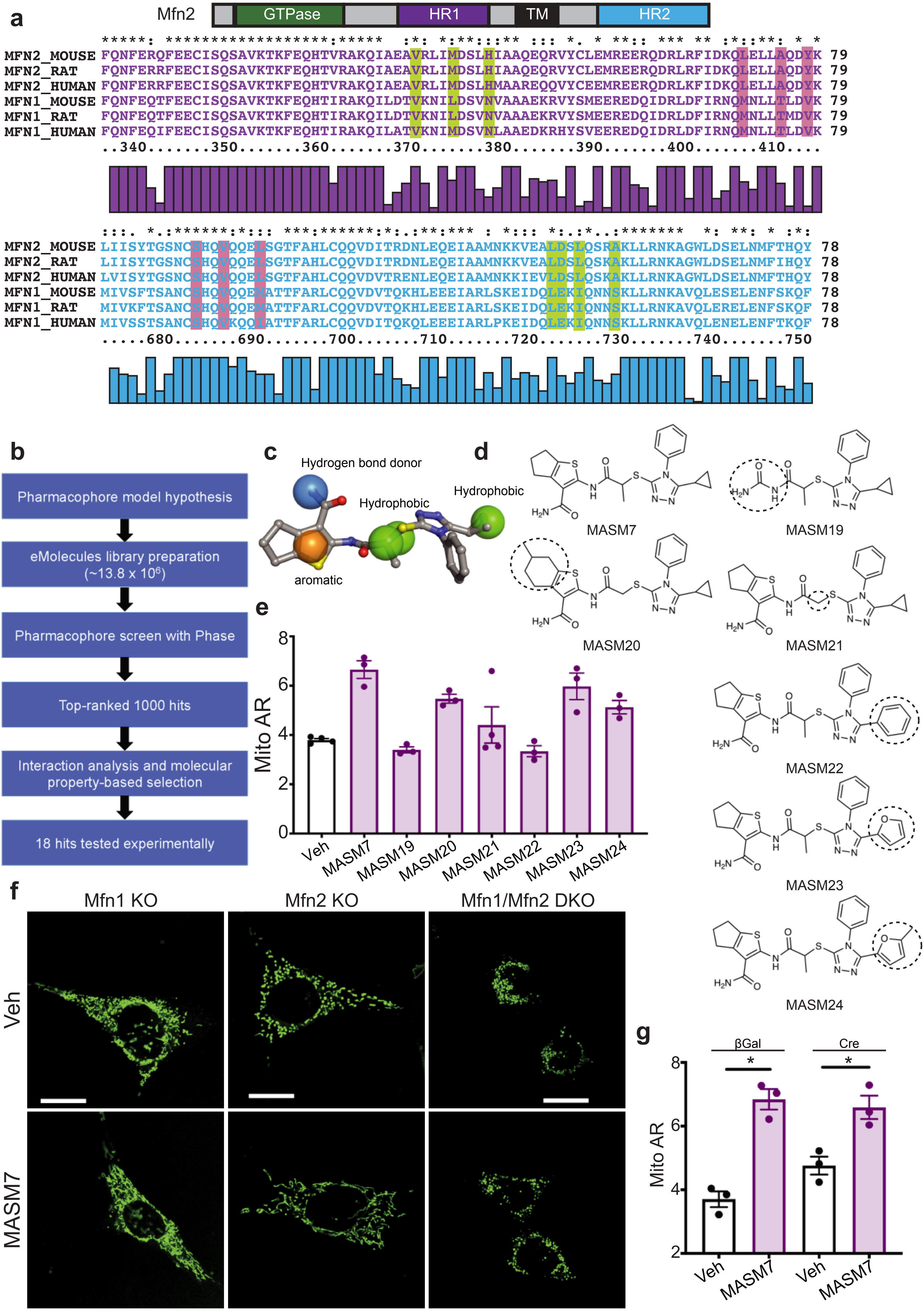
a
Mfn2
GTPase
HR1
TM
HR2
MFN2_MOUSE FQNFERQFEECISQSAVKTKFEQHTVRAKQIAEAVRLIMDSLHIAAQEQRVYCLEMREERQDRLRFIDKQLELLAQDYK 79
MFN2_RAT FQNFERRFEECISQSAVKTKFEQHTVRAKQIAEAVRLIMDSLHIAAQEQRVYCLEMREERQDRLRFIDKQLELLAQDYK 79
MFN2_HUMAN FQNFERRFEECISQSAVKTKFEQHTVRAKQIAEAVRLIMDSLHMAAREQQVYCEEMREERQDRLKFIDKQLELLAQDYK 79
MFN1_MOUSE FQNFEQTFEECISQSAVKTKFEQHTIRAKQILDTVKNILDSVNVAAAEKRVYSMEEREDQIDRLDFIRNQMNLLTLDVK 79
MFN1_RAT FQNFEQTFEECISQSAVKTKFEQHTIRAKQILDTVKNILDSVNVAAAEKRVYSMEEREDQIDRLDFIRNQMNLLTMDVK 79
MFN1_HUMAN FQNFEQIFEECISQSAVKTKFEQHTIRAKQILATVKNIMDSVNLAAEDKRHYSVEEREDQIDRLDFIRNQMNLLTLDVK 79
..340.......350.......360.......370.......380.......390.......400.......410....
MFN2_MOUSE LIISYTGSNCSHQVQQELSGTFAHLCQQVDITRDNLEQEIAAMNKKVEALDSLQSRAKLLRNKAGWLDSELNMFTHQY 78
MFN2_RAT LIISYTGSNCSHQVQQELSGTFAHLCQQVDITRDNLEQEIAAMNKKVEALDSLQSKAKLLRNKAGWLDSELNMFIHQY 78
MFN2_HUMAN LVISYTGSNCSHQVQQELSGTFAHLCQQVDVTRENLEQEIAAMNKKIEVLDSLQSKAKLLRNKAGWLDSELNMFTHQY 78
MFN1_MOUSE MIVSFTSANCSHQVQQEMATTFARLCQQVDVTQKHLEEEIARLSKEIDQLEKIQNNSKLLRNKAVQLESELENFSKQF 78
MFN1_RAT MIVKFTSANCSHQVQQEMATTFARLCQQVDVTQKHLEEEIARLSKEIDQLEKIQNNSKLLRNKAVQLERELENFSKQF 78
MFN1_HUMAN MIVSSTSANCSHQVKQQIATTFARLCQQVDITQKQLEEEIARLPKEIDQLEKIQNNSKLLRNKAVQLENELENFTKQF 78
.....680.......690.......700.......710.......720.......730.......740.......750
b
Pharmacophore model hypothesis
eMolecules library preparation (~13.8 x 10^6)
Pharmacophore screen with Phase
Top-ranked 1000 hits
Interaction analysis and molecular property-based selection
18 hits tested experimentally
c
Hydrogen bond donor
Hydrophobic
Hydrophobic
aromatic
d
MASM7
MASM19
MASM20
MASM21
MASM22
MASM23
MASM24
e
Mito AR
Veh
MASM7
MASM19
MASM20
MASM21
MASM22
MASM23
MASM24
f
Mfn1 KO
Mfn2 KO
Mfn1/Mfn2 DKO
Veh
MASM7
g
βGal
Cre
Mito AR
Veh
MASM7
Veh
MASM7

Supplementary Fig. 3. Pharmacophore-based discovery of mitofusin activators and lead MASM7.

a, Domain model of MFN2 showing GTPase (green), transmembrane region (black), HR1 (purple) and HR2 (blue). Sequences of MFN1 and MFN2 share high sequence homology between human, mouse and rat. HR1 and HR2 residues used in the pharmacophore hypothesis for the discovery of MASM7 and MFI8 demonstrate high sequence homology as highlighted in green and magenta, respectively. **b**, Schematic representation of the in silico-based screening strategy for the discovery of putative MASMs. **c**, MASM7 chemical structure fits the pharmacophore model criteria. **d,** Chemical structures of MASM7 analogues provide structure activity relationships. **e,** Quantification of Mito AR in MEFs. Cells were treated with MASM7 and analogues from d (1 μM, 2 h). Data represent mean ± SEM of three independent biological replicates. **f**, Confocal micrographs of *Mfn1* KO, *Mfn2* KO, *Mfn1/Mfn2* DKO MEFs. Cells were treated with MASM7 (1 μM, 2 h). Mitochondria were stained with Mitotracker green. Scale bar 20 μm. Each micrograph is representative of n=3 independent experiments. **g**, Quantification of Mito AR of *Drp1*$^{FLOX/FLOX}$ MEFs. Cells were treated with MASM7 (1 μM, 2 h). Data represent mean ± SEM of three independent biological replicates. Statistics were obtained using two-tailed unpaired t-test: *$p<0.05$, **$p<0.01$, ***$p<0.001$, ****$p<0.0001$. Source data are provided as a Source Data file.

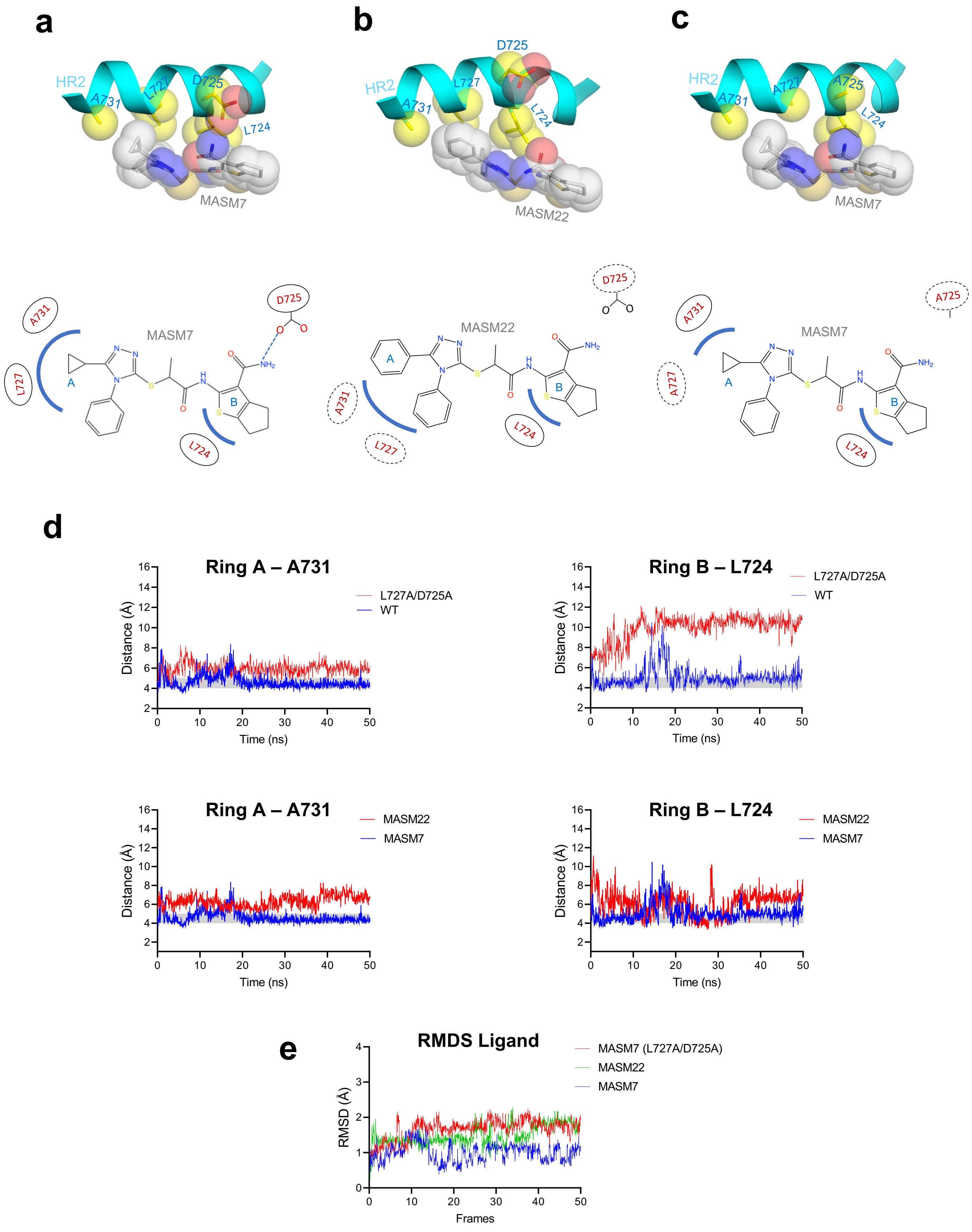

a
b
c
HR2
A731
L727
D725
L724
MASM7
MASM22
A727
A725
A
B
d
Ring A – A731
Ring B – L724
L727A/D725A
WT
MASM22
MASM7
Distance (Å)
Time (ns)
e
RMDS Ligand
MASM7 (L727A/D725A)
MASM22
MASM7
RMSD (Å)
Frames

Supplementary Fig. 4. Characterization of MASM7-MFN2 interaction using molecular docking and molecular dynamics.

a, Docked structure of MASM7 to MFN2-HR2. MASM7 and MFN2-HR2 interacting residues shown in spheres (top) and schematic of interaction (bottom), depicting hydrogen bond between D725 and MASM7 and several van der Waals contacts (blue line) with hydrophobic residues A731, L727 and L724. **b,** Docked structure of MASM22 (upper) and schematic of MASM22-HR2 residue interactions, showing disruption of interactions with D725, A731 and L727 (dashed lines). **c,** Docked structure of MASM7 to mutated MFN2-HR2L725A/L727A (upper) and schematic (bottom), showing loss of interaction with L725 and L727. **d,** Dynamics of interactions between MASM7/MASM22 and HR2-WT/HR2-L725A/L727A after MD simulations starting from the docking poses shown in a, b, c. The plots demonstrate in all cases loss of contacts between MASM22 and MFN2-HR2 or between MASM7 and mutated MFN2-HR2L725A/L727A (in red) compared to MASM7 or MFN2-HR2 WT (in blue), respectively. Ring distances shown are calculated from the geometric centers of MASM7 rings depicted in a to Cβ carbon of A731 or closest side-chain carbon of L724, respectively. **e,** RMSD of ligands during the MD simulation. Source data are provided as a Source Data file.

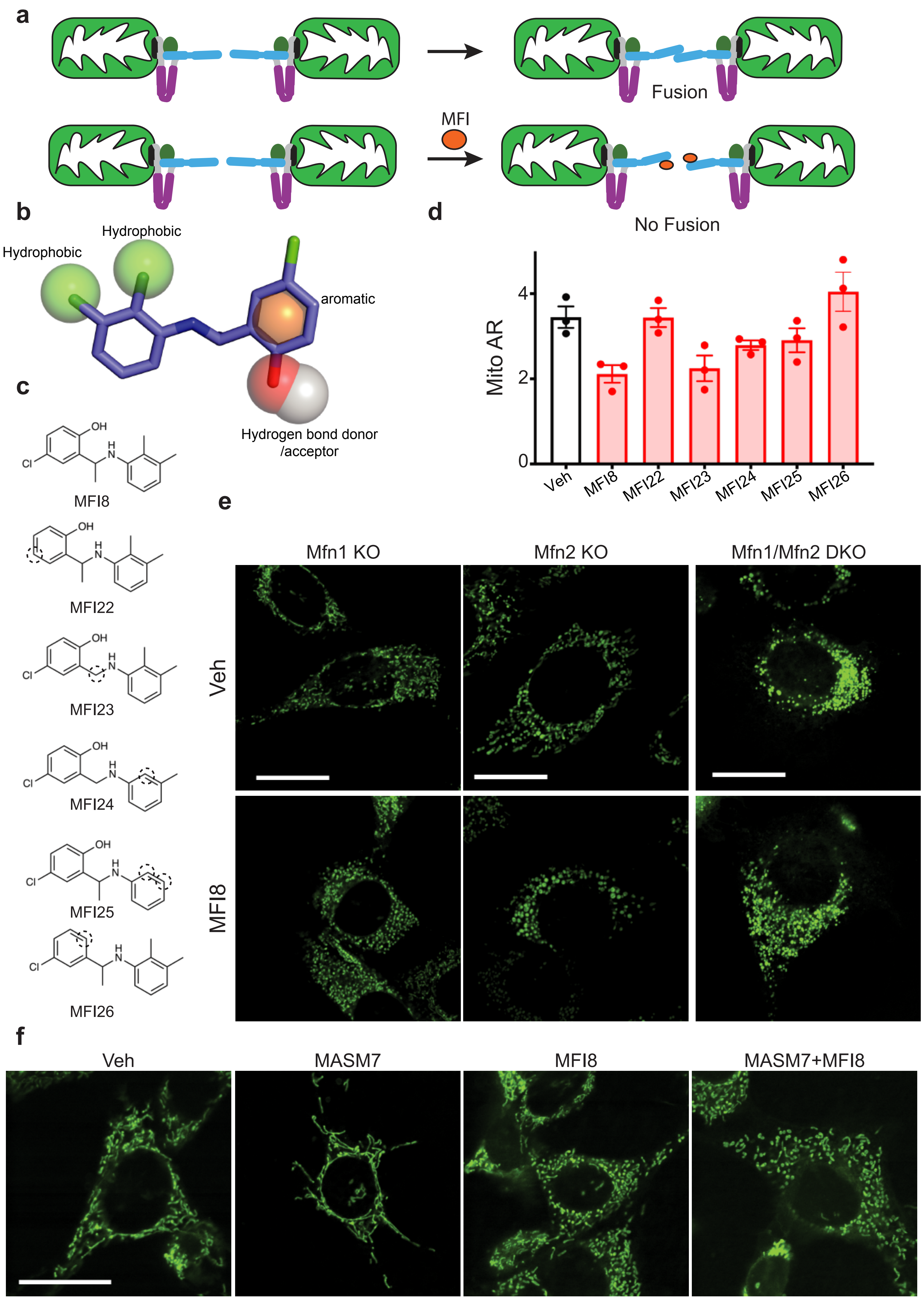
a
Fusion
MFI
No Fusion
b
Hydrophobic
Hydrophobic
aromatic
Hydrogen bond donor
/acceptor
c
MFI8
MFI22
MFI23
MFI24
MFI25
MFI26
d
Mito AR
Veh
MFI8
MFI22
MFI23
MFI24
MFI25
MFI26
e
Mfn1 KO
Mfn2 KO
Mfn1/Mfn2 DKO
Veh
MFI8
f
Veh
MASM7
MFI8
MASM7+MFI8

Supplementary Fig. 5. Pharmacophore-based discovery of mitofusin inhibitors and lead MFI8.

a, Cartoon depiction of the MFNs-mediated tethering of the outer mitochondrial membrane. Mitochondrial fusion inhibitor (MFI) potentially acts as protein-protein interaction inhibitor of MFNs targeting specific amino acids of HR2-HR2 interaction in the pro-tethering conformation to prevent mitochondrial fusion. **b,** MFI8 chemical structure fits pharmacophore model criteria. **c,** Chemical structures of MFI8 analogues provide structure activity relationships. **d**, Quantification of Mito AR of MEFs that were treated with MFI8 analogues (20 μM, 6 h). Data represent mean ± SEM of three independent biological replicates. **e**, Confocal micrographs of *Mfn1* KO, *Mfn2* KO, *Mfn1/Mfn2* DKO MEFs. Cells were treated with MFI8 (20 μM, 6 h). Mitochondria were stained with Mitotracker green. Scale bar 20 μm. Each micrograph is representative of n=3 independent experiments. **f**, Confocal micrographs of MEFs that were treated with MASM7 (1 μM), MFI8 (20 μM) or both for 6 h. Mitochondria were stained with Mitotracker green. Scale bar 20 μm. Each micrograph is representative of n=3 independent experiments. Source data are provided as a Source Data file.

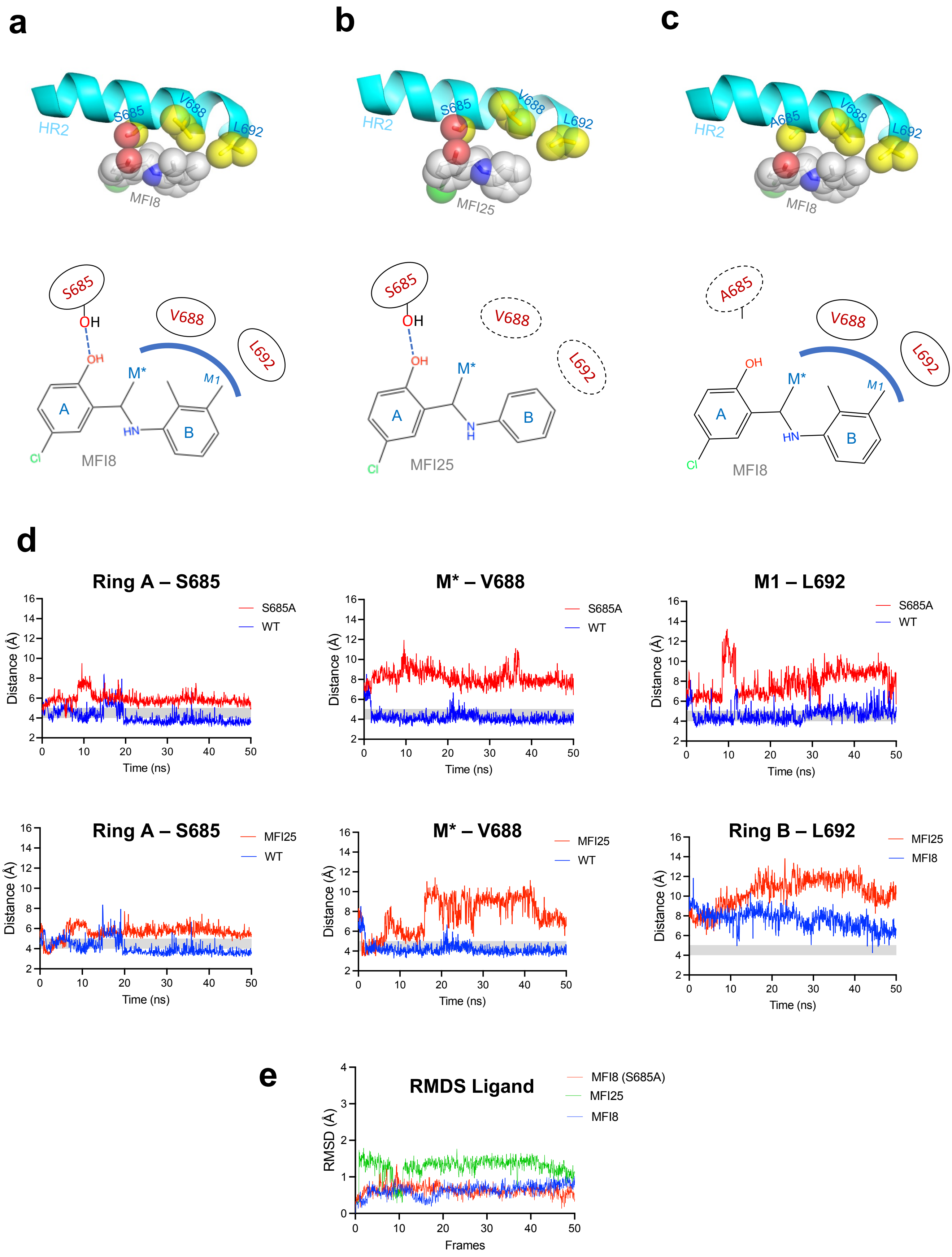

a
b
c
HR2
S685
V688
L692
A685
MFI8
MFI25
OH
M*
M1
A
B
HN
Cl
d
Ring A – S685
M* – V688
M1 – L692
Ring B – L692
S685A
WT
MFI25
MFI8
Distance (Å)
Time (ns)
e
RMDS Ligand
MFI8 (S685A)
RMSD (Å)
Frames

Supplementary Fig. 6. Characterization of MFI8-MFN2 interaction using molecular docking and molecular dynamics.

a, Docked structure of MFI8 to MFN2-HR2. MFI8 and MFN2-HR2 interacting residues are shown in spheres (top) and schematic of the interaction (bottom), depicting hydrogen bond between MFI8 hydroxyl and S685 and van der Waals interactions (blue line) with hydrophobic residues V688 and L692. **b,** Docked structure of MFI25 to MFN2-HR2 (upper) and schematic of MFI25-HR2 residue interactions, showing disruption of van der Waals contacts to residues V688 and L692 (dashed lines). **c,** Docked structure of MFI8 to mutant MFN2-HR2S685A (upper) and schematic (bottom), showing disruption of hydrogen bond of MFI8 to S685. **d,** Dynamics of interactions between MFI8/MFI25 and HR2-WT/HR2-S685A after MD simulations starting from the docking poses shown in a, b, c. The plots demonstrate in all cases loss of contacts between MFI25 and MFN2-HR2 or between MFI8 and mutated MFN2-HR2S685A (in red) compared to MFI8 or MFN2-HR2WT (in blue), respectively. Atomic distances to S685 are to the Cβ carbon. Distance from MFI8 atoms M* and M1 are to the closest side-chain carbon of V688 or L692, respectively. Ring distances shown are calculated from the geometric centers of MFI8 rings depicted in a. **e,** RMSD of ligands during the MD simulation. Source data are provided as a Source Data file.

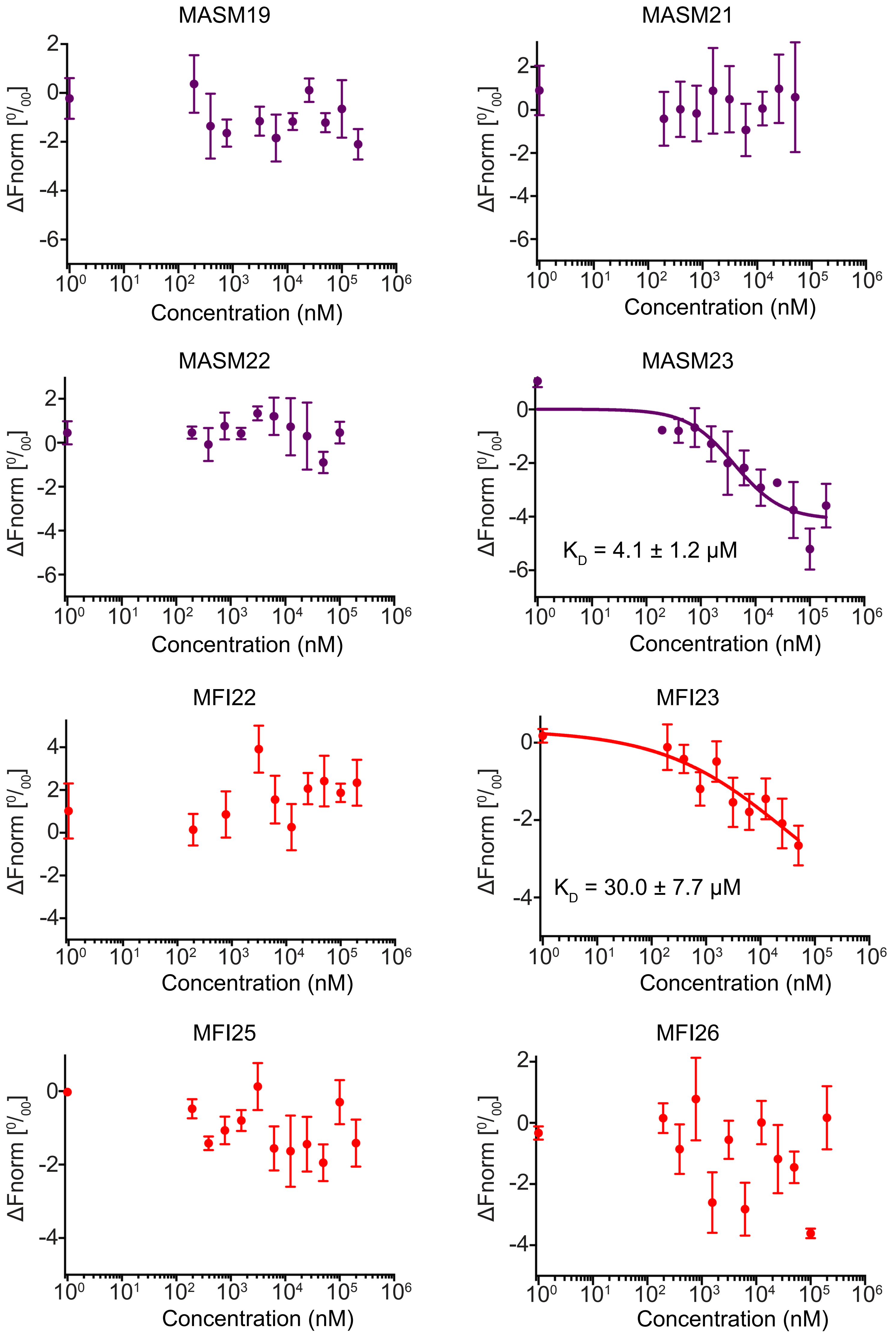
MASM19
MASM21
MASM22
MASM23
K_D = 4.1 ± 1.2 μM
MFI22
MFI23
K_D = 30.0 ± 7.7 μM
MFI25
MFI26
ΔFnorm [0/00]
Concentration (nM)

Supplementary Fig. 7. Microscale thermophoresis to determine direct interaction of MASM7 and MFI8 analogues to the MFN2-HR2 domain.

Plots of mean corrected normalized fluorescence (ΔF_{norm}: F_{nomr} bound - F_{norm} unbound) from MST signal analysis of titrations of MFN2-HR2 with indicated compounds. Kd values were obtained when possible from non-linear regression fits of normalized data to a three or four parameter logistic curve, using GraphPad Prism 8. Data represent mean ± SEM from three replicate experiments. Source data are provided as a Source Data file.

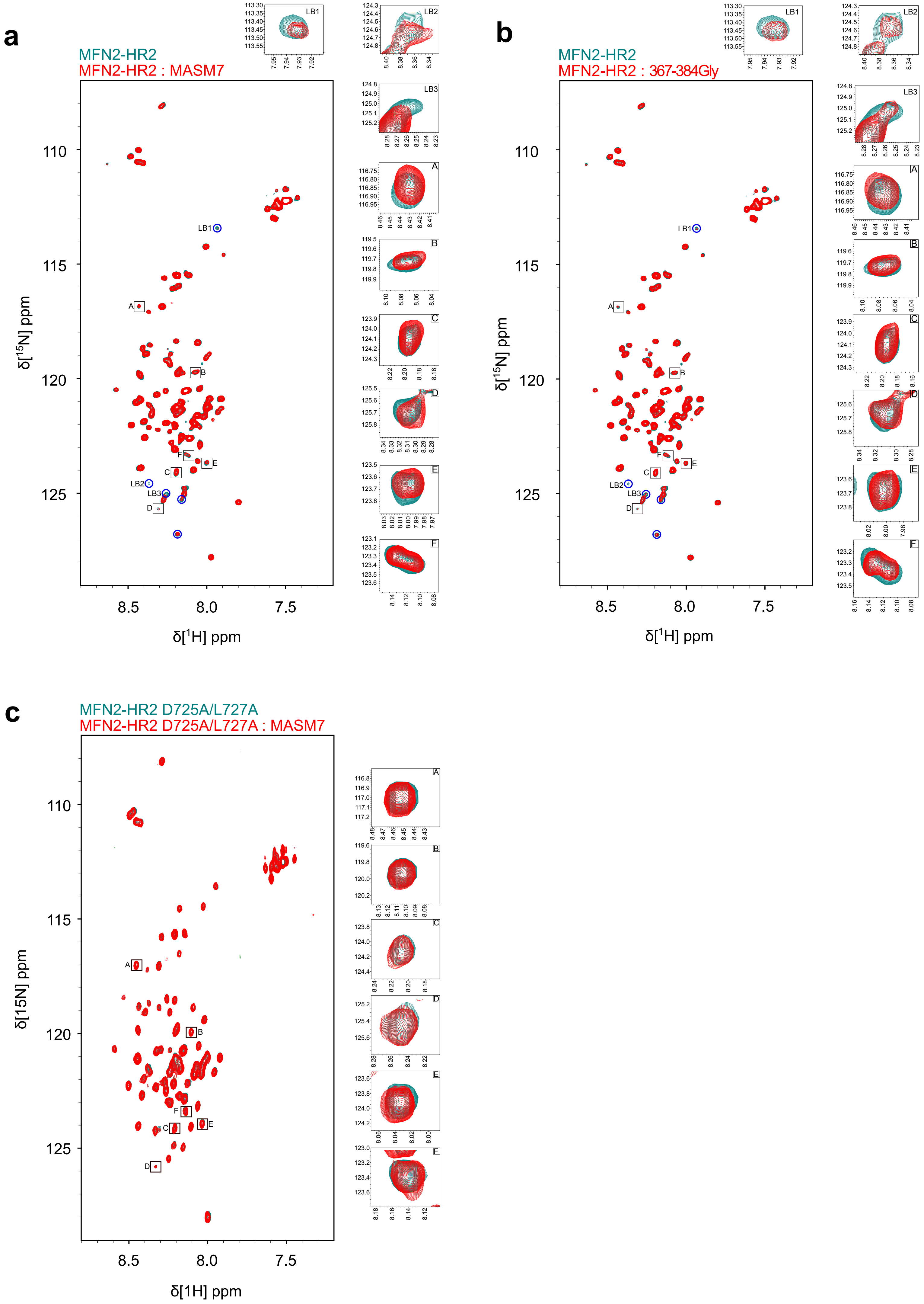

a
MFN2-HR2
MFN2-HR2 : MASM7
δ[15N] ppm
δ[1H] ppm
LB1
LB2
LB3
A
B
C
D
E
F
b
MFN2-HR2
MFN2-HR2 : 367-384Gly
δ[15N] ppm
δ[1H] ppm
c
MFN2-HR2 D725A/L727A
MFN2-HR2 D725A/L727A : MASM7
δ[15N] ppm
δ[1H] ppm

Supplementary Fig. 8. MASM7 interacts specifically with the HR2 domain of MFN2. **a**, Comparison of the ^{1}H-^{15}N HSQC spectra of MFN2-HR2 (50 μM) with (red cross-peaks) and without (teal cross-peaks) MASM7 (200 μM). Examples of the observed peak broadening and shifting upon addition of MASM7 are highlighted. **b**, Comparison of the ^{1}H-^{15}N HSQC spectra of MFN2-HR2 (50 μM) with (red cross-peaks) and without (teal cross-peaks) 367-384Gly peptide (200 μM). Same cross peaks as shown in (a) are highlighted. **c**, Comparison of the ^{1}H-^{15}N HSQC spectra of MFN2-HR2 D725A/L727A mutant (50 μM) with (red cross-peaks) and without (teal cross-peaks) MASM7 (200 μM). Same cross peaks as shown in (a) are highlighted. Data are representative of two independent biological replicates.

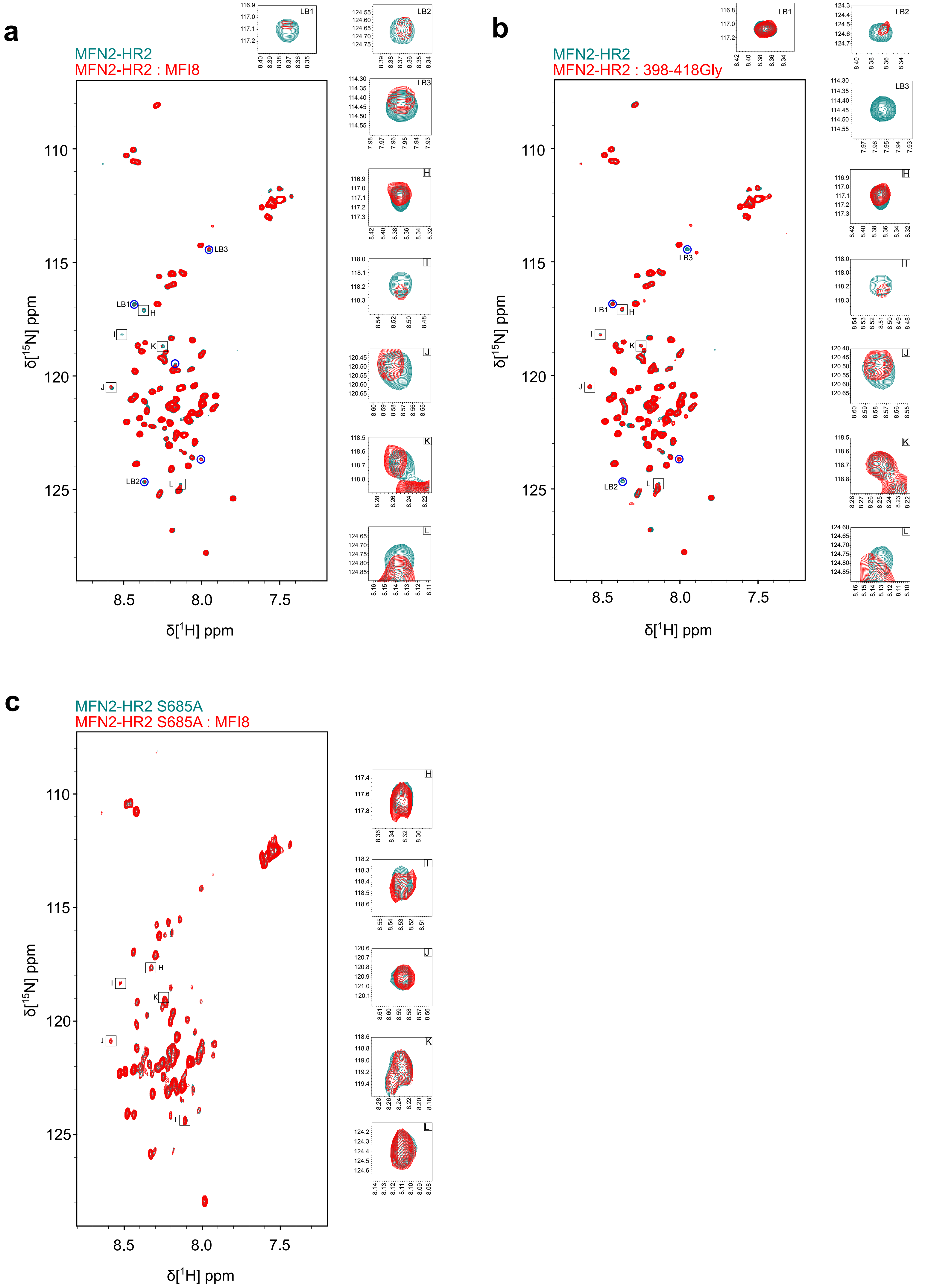
a
MFN2-HR2
MFN2-HR2 : MFI8
δ[15N] ppm
δ[1H] ppm
110
115
120
125
8.5
8.0
7.5
LB1
LB2
LB3
H
I
J
K
L
b
MFN2-HR2
MFN2-HR2 : 398-418Gly
δ[15N] ppm
δ[1H] ppm
LB1
LB2
LB3
H
I
J
K
L
c
MFN2-HR2 S685A
MFN2-HR2 S685A : MFI8
δ[15N] ppm
δ[1H] ppm
H
I
J
K
L

Supplementary Fig. 9. MFI8 interacts specifically with the HR2 domain of MFN2.

a, Comparison of the ^{1}H-^{15}N HSQC spectra of MFN2-HR2 (50 μM) with (red cross-peaks) and without (teal cross-peaks) MFI8 (200 μM). Examples of the observed peak broadening and shifting upon addition of MFI8 are highlighted. **b**, Comparison of the ^{1}H-^{15}N HSQC spectra of MFN2-HR2 (50 μM) with (red cross-peaks) and without (teal cross-peaks) 398-418Gly peptide (200 μM). Same cross peaks as shown in (a) are highlighted. **c**, Comparison of the ^{1}H-^{15}N HSQC spectra of MFN2-HR2 S685A mutant (50 μM) with (red cross-peaks) and without (teal cross-peaks) MFI8 (200 μM). Same cross peaks as shown in (a) are highlighted. Data are representative of two independent biological replicates.

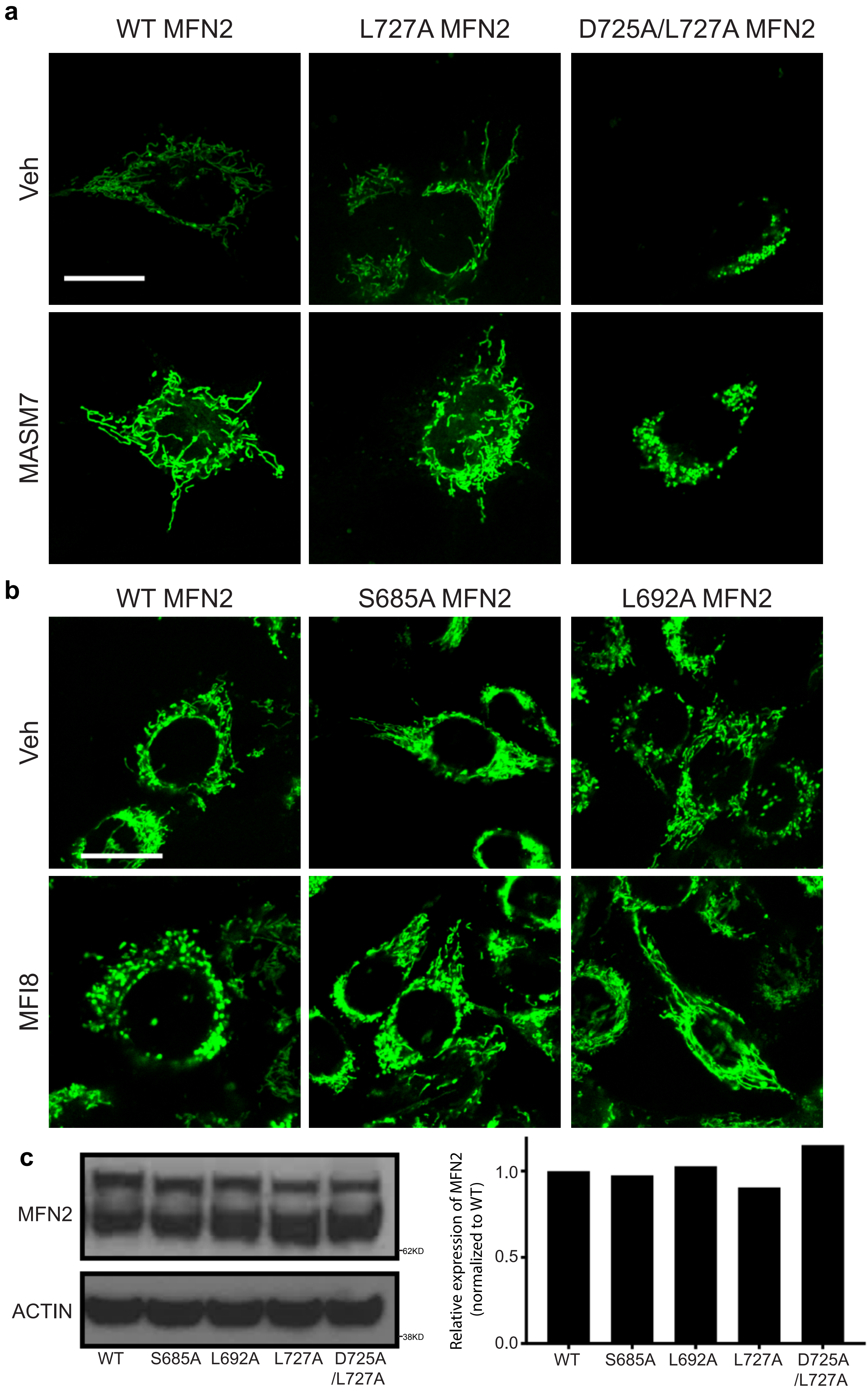

a
WT MFN2
L727A MFN2
D725A/L727A MFN2
Veh
MASM7
b
WT MFN2
S685A MFN2
L692A MFN2
Veh
MFI8
c
MFN2
ACTIN
62KD
38KD
WT
S685A
L692A
L727A
D725A /L727A
Relative expression of MFN2 (normalized to WT)
1.0
0.5
0.0
WT
S685A
L692A
L727A
D725A /L727A

Supplementary Fig. 10. Confocal micrographs of cells that were reconstituted with MFN2 mutants.

a, Confocal micrographs of *Mfn1/Mfn2* DKO MEFs reconstituted with WT, L727A and D725A/L727A mutants of *Mfn*2. Mitochondria were stained with Mitotracker green. Cells were treated with MASM7 (1 μM, 6 h). Scale bar 20 μm. Each micrograph is representative of n=3 independent experiments. **b**, Confocal micrographs of *Mfn1/Mfn2* DKO MEFs reconstituted with WT, S685A and L692A *Mfn*2. Mitochondria were stained with Mitotracker green. Cells were treated with MFI8 (20 μM, 6 h). Scale bar 20 μm. Each micrograph is representative of n=3 independent experiments. **c**, Western blot analysis of expression levels of alanine-substituted MFN2 mutants that were used in a and b (left) and quantification of alanine-substituted MFN2 mutants in relation to the reconstituted WT *Mfn*2 (right). Source data are provided as a Source Data file.

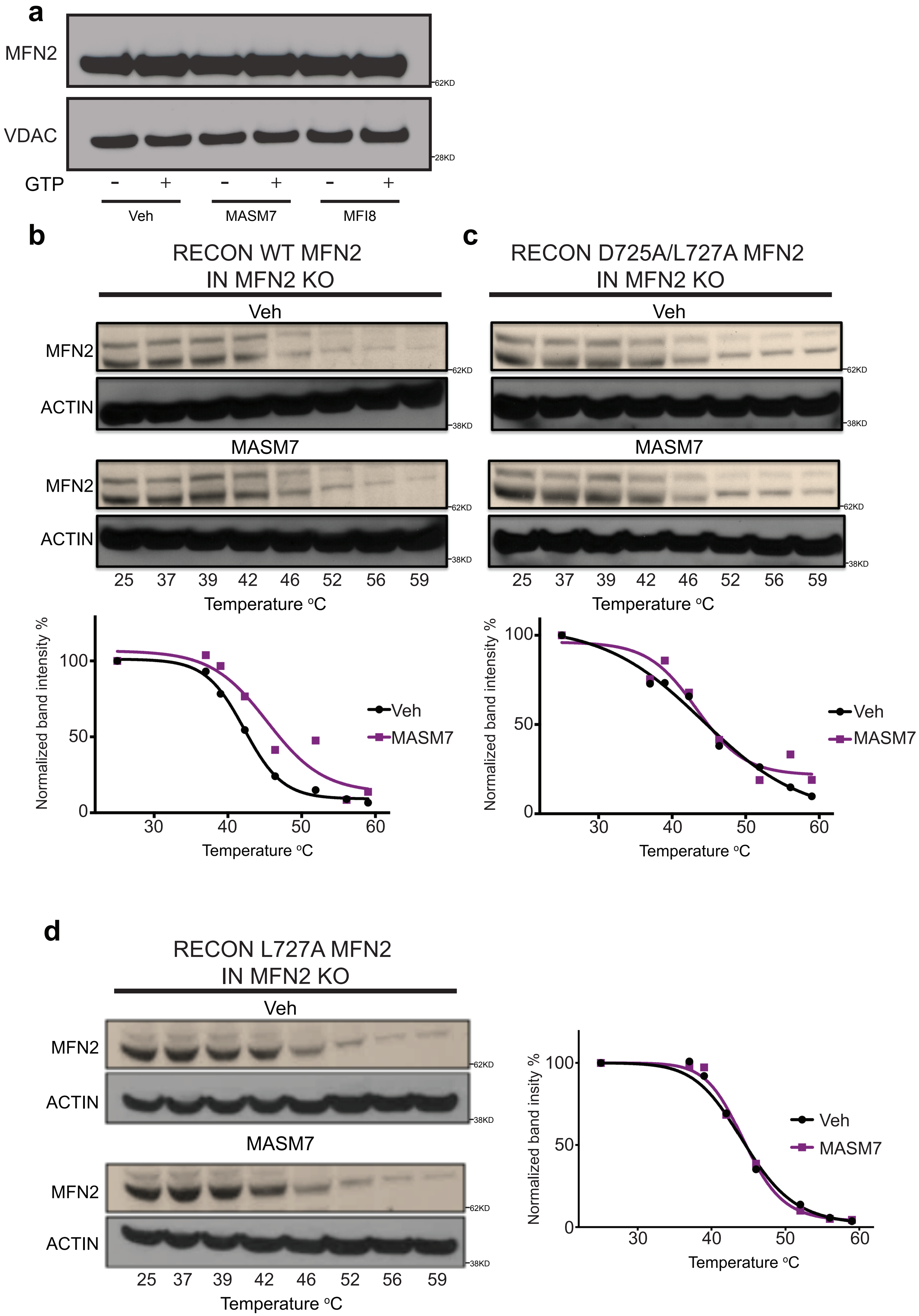
a
MFN2
VDAC
62KD
28KD
GTP - + - + - +
Veh
MASM7
MFI8
b
RECON WT MFN2
IN MFN2 KO
c
RECON D725A/L727A MFN2
IN MFN2 KO
Veh
MASM7
MFN2
ACTIN
62KD
38KD
25 37 39 42 46 52 56 59
Temperature °C
Normalized band intensity %
Veh
MASM7
d
RECON L727A MFN2
IN MFN2 KO
Normalized band insity %

Supplementary Fig. 11. MASM7 engages MFN2 in cells.

a, Western blot of MFN2. Isolated mitochondria were treated with GTP, MASM7 and MFI8 for 30 min. VDAC was used a loading control for the mitochondrial fraction. Blot is representative of n=2 independent experiments. **b**, Cellular engagement (CETSA) of MFN2 by MASM7 in *Mfn2* KO MEFs reconstituted with WT *Mfn*2. A representative blot from two independent experiments is shown (top). Quantification of temperature-dependent normalized MFN2 levels obtained by densitometry with corresponding fitted curves. Data represent mean ± SEM of two independent biological replicates (bottom). **c**, Cellular engagement (CETSA) of MFN2 by MASM7 in *Mfn2* KO MEFs reconstituted with D725A/L727A *Mfn*2. A representative blot from two independent experiments is shown (top). Quantification of temperature-dependent normalized MFN2 levels obtained by densitometry with corresponding fitted curves. Data represent mean ± SEM of two independent biological replicates (bottom). **d**, Cellular engagement (CETSA) of MFN2 by MASM7 in *Mfn2* KO MEFs reconstituted with L727A *Mfn*2. A representative blot from two independent experiments is shown (left). Quantification of temperature-dependent normalized MFN2 levels obtained by densitometry with corresponding fitted curves. Data represent mean ± SEM of two independent biological replicates (right). Source data are provided as a Source Data file.

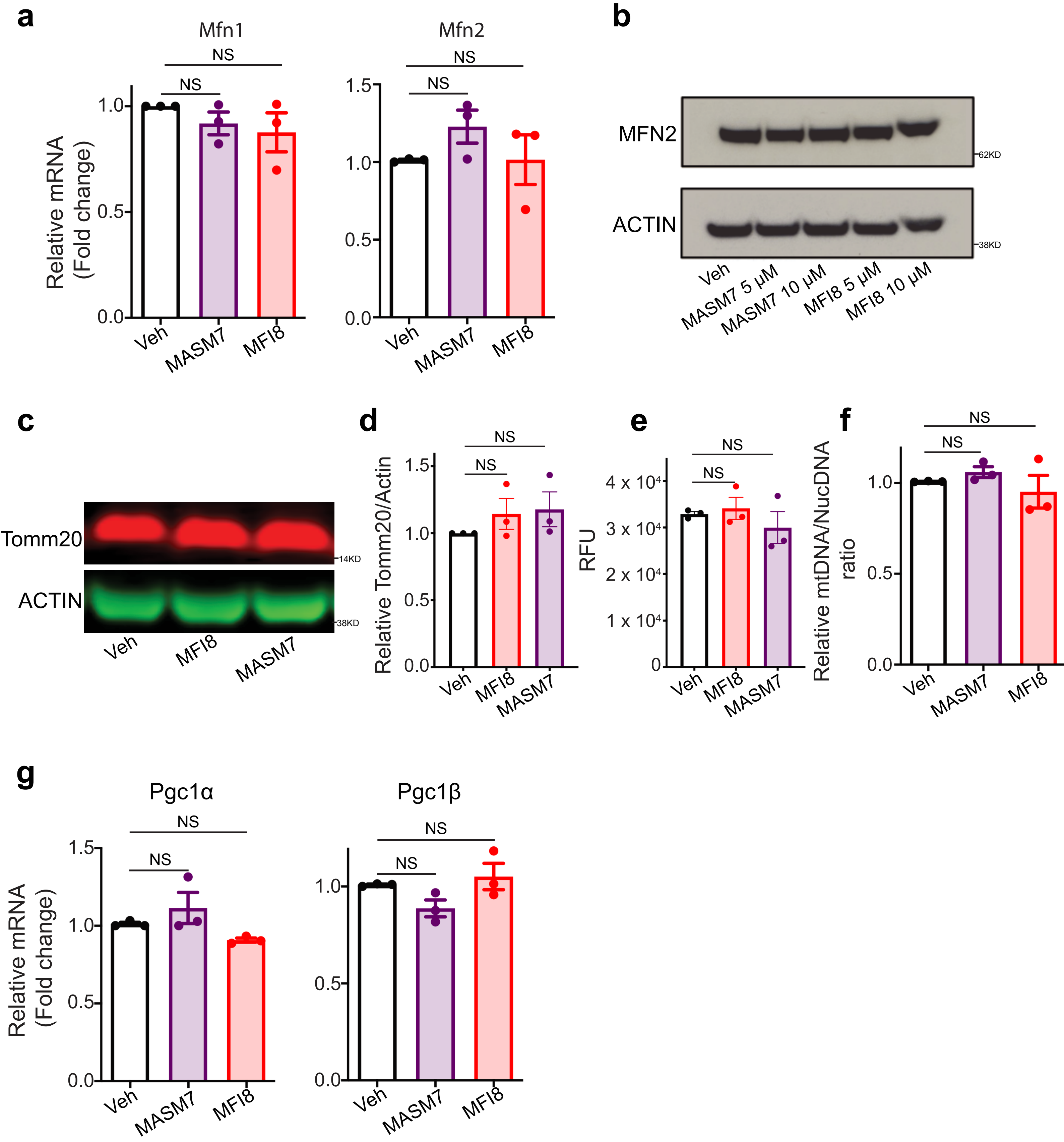
a
Mfn1
Mfn2
NS
Relative mRNA (Fold change)
Veh
MASM7
MFI8
b
MFN2
ACTIN
62KD
38KD
Veh
MASM7 5 μM
MASM7 10 μM
MFI8 5 μM
MFI8 10 μM
c
Tomm20
ACTIN
14KD
38KD
d
Relative Tomm20/Actin
e
RFU
4 x 10⁴
3 x 10⁴
2 x 10⁴
1 x 10⁴
f
Relative mtDNA/NucDNA ratio
g
Pgc1α
Pgc1β

Supplementary Fig. 12. MASM7 and MFI8 do not change Mfn1 and Mfn2 gene expression or mitochondrial biogenesis.

a, Gene expression analysis of *Mfn1* and *Mfn2* genes. MEFs treated with MASM7 (1 μM) or MFI8 (20 μM) for 6 h. Data represent mean ± SEM of three independent biological replicates. **b**, Western blot of MFN2. MEFs treated with MASM7 or MFI8 at the indicated concentrations for 6 h. **c**, Western blot of Tomm20. MEFs treated with MASM7 (1 μM) or MFI8 (20 μM) for 6 h. **d**, Quantification of (c). Data represent mean ± SEM of three independent biological replicates. **e**, Quantification of fluorescent intensity of mitotracker green. MEFs treated with MASM7 (1 μM) or MFI8 (20 μM) for 6 h. Data represent mean ± SEM of three independent biological replicates. **f**, Relative mitochondrial (mt) DNA levels. MEFs treated with MASM7 (1 μM) or MFI8 (20 μM) for 6 h. Data represent ±SEM of three independent biological replicates. **g**, Gene expression analysis of *Pgc1α* and *Pgc1β* genes. MEFs treated with MASM7 (1 μM) or MFI8 (20 μM) for 6 h. Data represent ±SEM of three independent biological replicates. Source data are provided as a Source Data file.

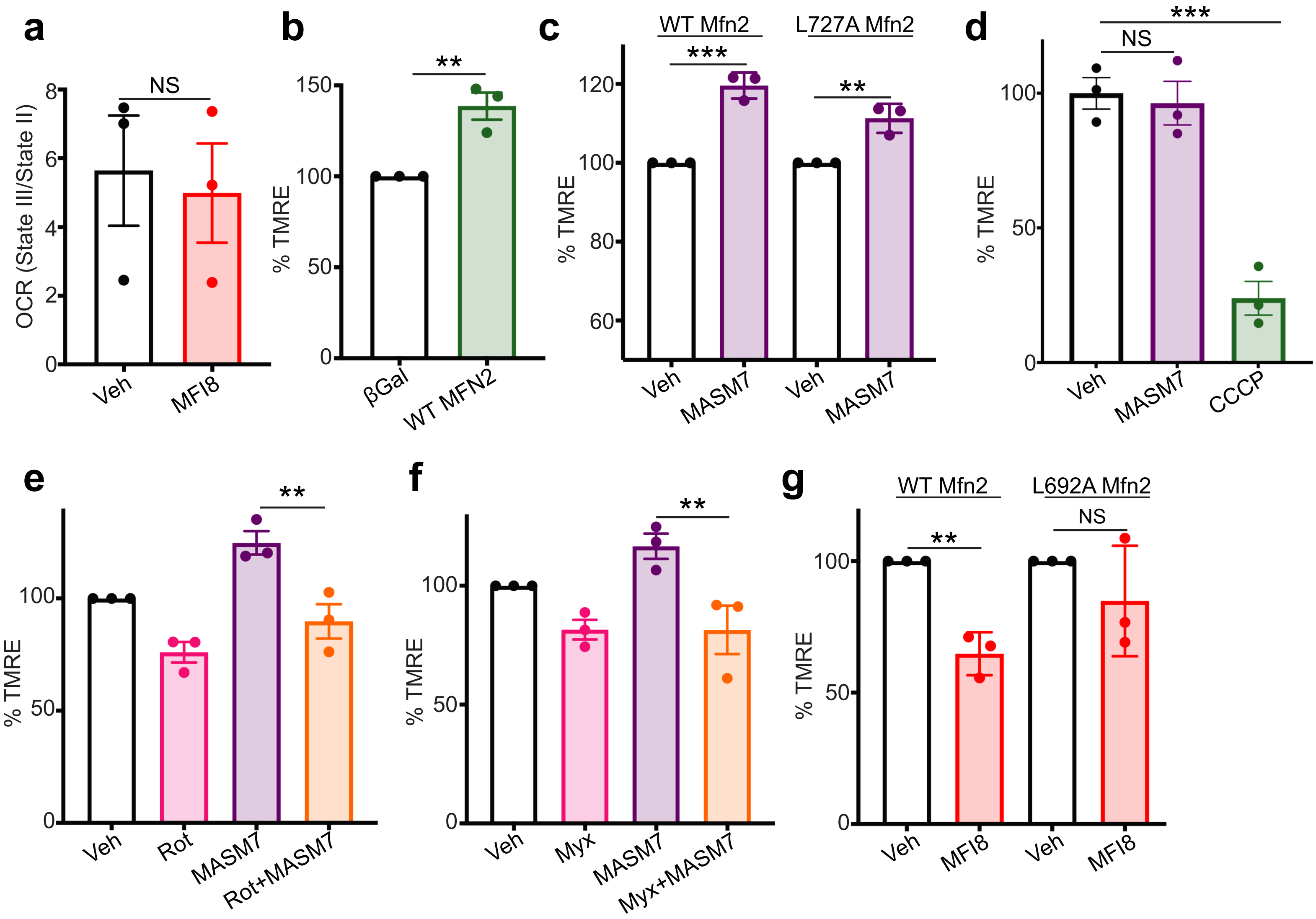
a
OCR (State III/State II)
NS
Veh
MFI8
b
% TMRE
**
βGal
WT MFN2
c
WT Mfn2
L727A Mfn2
***
**
% TMRE
Veh
MASM7
Veh
MASM7
d
***
NS
% TMRE
Veh
MASM7
CCCP
e
**
% TMRE
Veh
Rot
MASM7
Rot+MASM7
f
**
% TMRE
Veh
Myx
MASM7
Myx+MASM7
g
WT Mfn2
L692A Mfn2
**
NS
% TMRE
Veh
MFI8
Veh
MFI8

Supplementary Fig. 13. MASM7 and MFI8 alter mitochondrial functionality.

a, OCR ratio (state III/state II) in isolated mitochondria from murine cardiomyocytes. Mitochondria were treated with MFI8 (20 μM, 30 min). Data represent mean ± SEM of three independent biological replicates. **b**, Mitochondrial membrane potential assay in MEFs using TMRE as a readout. *Mfn1/Mfn2* DKO MEFs were reconstituted with WT MFN2 or βGal. Data represent ±SEM of three independent biological replicates. **c**, Mitochondrial membrane potential assay in MEFs using TMRE as a readout. *Mfn1/Mfn2* DKO MEFs were reconstituted with WT *Mfn*2 or L727A *Mfn*2. Cells were treated with MASM7 (1 μM, 6 h). Data represent mean ± SEM of three independent biological replicates. **d**, Mitochondrial membrane potential assay in isolated mitochondria from murine cardiomyocytes. Mitochondria were treated with MASM7 (10 μM, 30 min) or CCCP (30 μM, 30 min). CCCP was used a positive control. Data represent mean ± SEM of three independent biological replicates**. e,** Mitochondrial membrane potential assay in MEFs using TMRE as a readout. Cells were treated with MASM7 (1 μM, 6 h) and rotenone (Rot; 10 μM, 30 min). Data represent mean ± SEM of three independent biological replicates. **f**, Mitochondrial membrane potential assay in MEFs using TMRE as a readout. Cells were treated with MASM7 (1 μM, 6 h) and myxothiazol (Myx; 10 μM, 30 min). Data represent mean ± SEM of three independent biological replicates. **g**, Mitochondrial membrane potential assay in MEFs using TMRE as a readout. *Mfn1/Mfn2* DKO MEFs were reconstituted with WT *Mfn*2 or L692A *Mfn*2. Cells were treated with MFI8 (20 μM, 6 h). Data represent mean ± SEM of three independent biological replicates. Statistics were obtained using two-tailed unpaired t-test for panels b, c, g or one way ANOVA for panels d, e, f: *$p<0.05$, **$p<0.01$, ***$p<0.001$, ****$p<0.0001$. Source data are provided as a Source Data file.

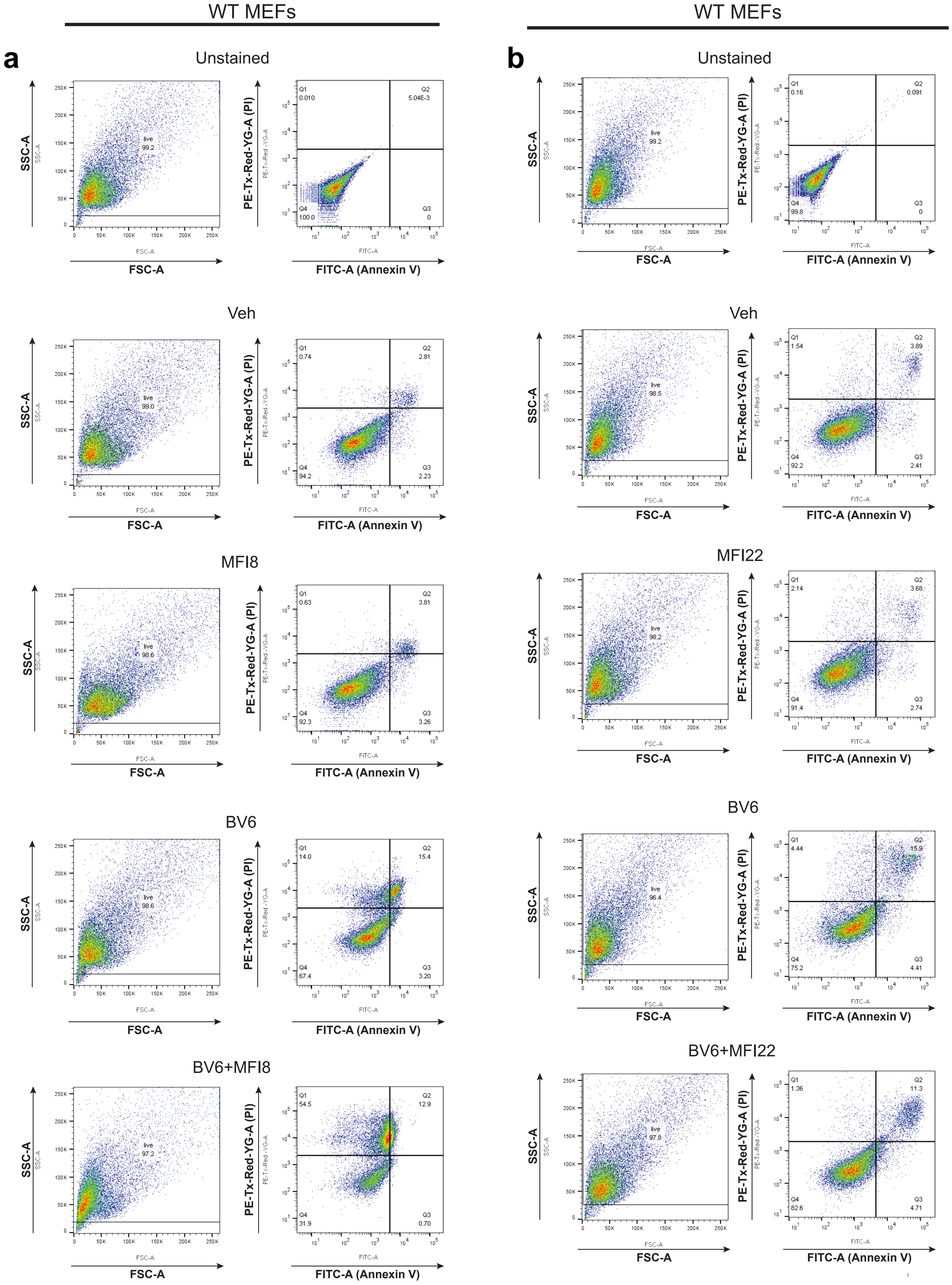

WT MEFs
a
Unstained
SSC-A
FSC-A
live 99.2
PE-Tx-Red-YG-A (PI)
FITC-A (Annexin V)
Q1 0.010
Q2 5.04E-3
Q3 0
Q4 100.0
Veh
live 99.0
Q1 0.74
Q2 2.81
Q3 2.23
Q4 94.2
MFI8
live 98.6
Q1 0.63
Q2 3.81
Q3 3.26
Q4 92.3
BV6
live 98.6
Q1 14.0
Q2 15.4
Q3 3.20
Q4 67.4
BV6+MFI8
live 97.2
Q1 54.5
Q2 12.9
Q3 0.70
Q4 31.9
WT MEFs
b
Unstained
live 99.2
Q1 0.16
Q2 0.091
Q3 0
Q4 99.8
Veh
live 98.5
Q1 1.54
Q2 3.89
Q3 2.41
Q4 92.2
MFI22
live 98.2
Q1 2.14
Q2 3.68
Q3 2.74
Q4 91.4
BV6
live 96.4
Q1 4.44
Q2 15.9
Q3 4.41
Q4 75.2
BV6+MFI22
live 97.8
Q1 1.36
Q2 11.3
Q3 4.71
Q4 82.6

Supplementary Fig. 14. Gating strategy used for Annexin V/PI FACS experiments. a, **b**, Staining of WT MEFs. The right plots demonstrate the gating for FSC-A/SSC-A, while the left plots show Annexin V and propidium iodide (PI) positive cells according to the described treatments.

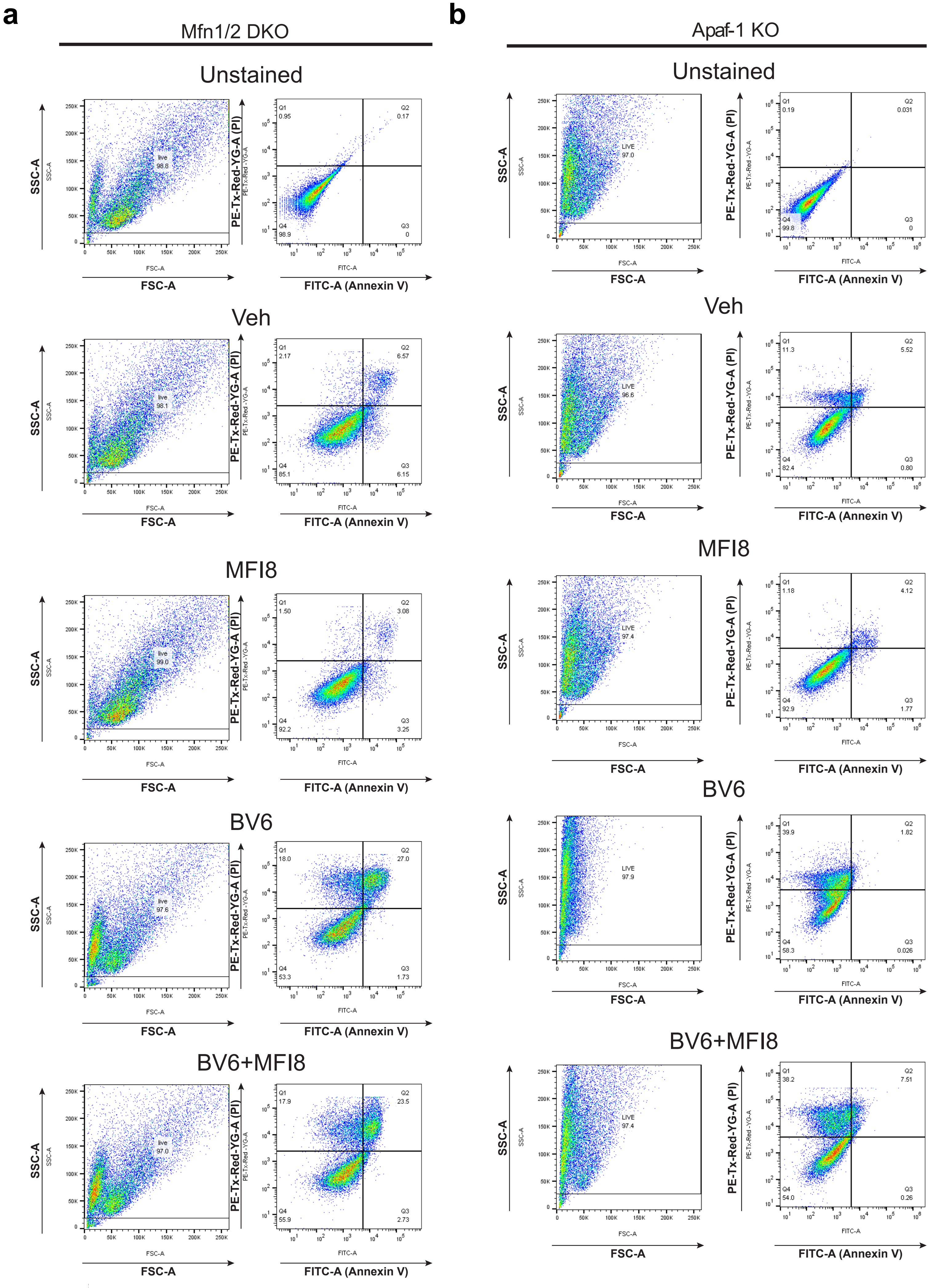

a
Mfn1/2 DKO
b
Apaf-1 KO
Unstained
Veh
MFI8
BV6
BV6+MFI8
SSC-A
FSC-A
PE-Tx-Red-YG-A (PI)
FITC-A (Annexin V)
live 98.8
Q1 0.95
Q2 0.17
Q4 98.9
Q3 0
live 98.1
Q1 2.17
Q2 6.57
Q4 85.1
Q3 6.15
live 99.0
Q1 1.50
Q2 3.08
Q4 92.2
Q3 3.25
live 97.6
Q1 18.0
Q2 27.0
Q4 53.3
Q3 1.73
live 97.0
Q1 17.9
Q2 23.5
Q4 55.9
Q3 2.73
LIVE 97.0
Q1 0.19
Q2 0.031
Q4 99.8
Q3 0
LIVE 96.6
Q1 11.3
Q2 5.52
Q4 82.4
Q3 0.80
LIVE 97.4
Q1 1.18
Q2 4.12
Q4 92.9
Q3 1.77
LIVE 97.9
Q1 39.9
Q2 1.82
Q4 58.3
Q3 0.026
LIVE 97.4
Q1 38.2
Q2 7.51
Q4 54.0
Q3 0.26

Supplementary Fig. 15. Gating strategy used for Annexin V/PI FACS experiments. a, Staining of *Mfn1/Mfn2* DKO MEFs. **b**, Staining of *Apaf*-1 KO MEFs. The right plots demonstrate the gating for FSC-A/SSC-A, while left plots show Annexin V and propidium iodide (PI) positive cells according to the described treatments.

a

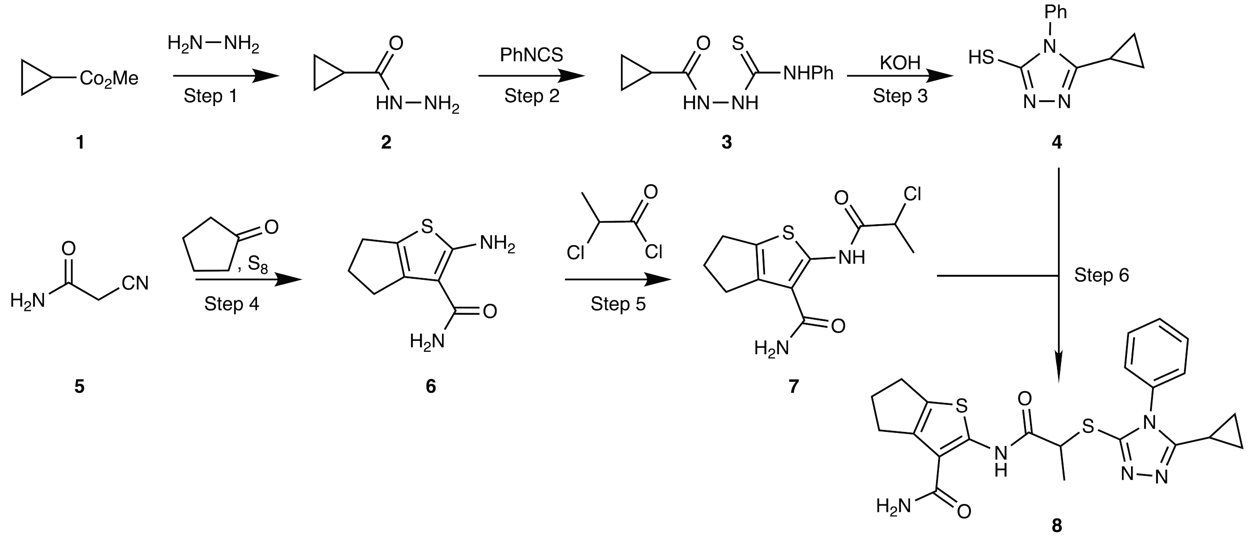

b

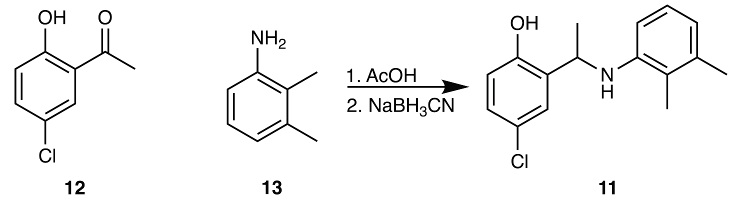

Supplementary Fig. 16. Synthetic schemes for MASM7 (a) and MFI8(b).

Supplementary Table 1. Primers used for the mutagenesis studies.

S685A (adeno)	5'-CTGGCTCCAACTGCGCCCACCAAGTCCAGC-3
L692A (adeno)	5'-CCAAGTCCAGCAGGAAGCGTCTGGGACCTTTGC-3'
S685A (NMR)	5'-TGGGTAGCAACTGCGCCCACCAGGTGCAGC-3'
L692A (NMR)	5'-AGGTGCAGCAAGAGGCGAGCGGCACCTTCG-3'
D725A/L727A	5'-AAGAAAATTGAAGTTCTGGCCAGCGCGCAAAGCAAGGCGAAACTG-3'
L727A	5'-AATTGAAGTTCTGGACAGCGCGCAAAGCAAGGCGAAACT-3'

Supplementary Table 2. Primers used for the mitochondrial biogenesis studies.

Gene	Forward	Reverse
Rpl139	CAAAATCGCCCTATTCCTCA	AGACCCAGCTTCGTTCTCCT
MFN1	GACCGAAGGGTCAGATGAAA	TCCAGCTCTGTGGTGACATC
MFN2	CCTGGATGCTGATGTGTTTG	CCAGCTCATCCACCAGAAAG
Ppargc1a	TGATGTGAATGACTTGGATACAGACA	GCTCATTGTTGTACTGGTTGGATATG
Ppargc1b	GGGAAAGGGACCAGACATAATC	GCGGAAGCAGATGGTAAGATAA

Supplementary Table 3. Primers used for the determination of mtDNA and genomic DNA.

Gene	Forward	Reverse
mt DNA (*Nd2*)	CCTATCACCCTTGCCATCAT	GAGGCTGTTGCTTGTGTGAC
gn DNA (*Pecam1*)	ATGGAAAGCCTGCCATCATG	TCCTTGTTGTTCAGCATCAC
