# Supplementary material for: Modulating mitofusins to control mitochondrial function and signaling
Source: Nat Commun. 2022 Jul 7;13:3775. doi: 10.1038/s41467-022-31324-1 (PMC9262907; doi:10.1038/s41467-022-31324-1)
Supplement: Supplementary file 3 — Reporting Summary [file 41467_2022_31324_MOESM3_ESM.pdf]

nature portfolio

Corresponding author(s): Evripidis Gavathiotis

Last updated by author(s): May 16, 2022

Reporting Summary

Nature Portfolio wishes to improve the reproducibility of the work that we publish. This form provides structure for consistency and transparency in reporting. For further information on Nature Portfolio policies, see our Editorial Policies and the Editorial Policy Checklist.

Statistics

For all statistical analyses, confirm that the following items are present in the figure legend, table legend, main text, or Methods section.

n/a	Confirmed	
☐	☒	The exact sample size (*n*) for each experimental group/condition, given as a discrete number and unit of measurement
☐	☒	A statement on whether measurements were taken from distinct samples or whether the same sample was measured repeatedly
☐	☒	The statistical test(s) used AND whether they are one- or two-sided<br>*Only common tests should be described solely by name; describe more complex techniques in the Methods section.*
☒	☐	A description of all covariates tested
☐	☒	A description of any assumptions or corrections, such as tests of normality and adjustment for multiple comparisons
☐	☒	A full description of the statistical parameters including central tendency (e.g. means) or other basic estimates (e.g. regression coefficient) AND variation (e.g. standard deviation) or associated estimates of uncertainty (e.g. confidence intervals)
☐	☒	For null hypothesis testing, the test statistic (e.g. *F*, *t*, *r*) with confidence intervals, effect sizes, degrees of freedom and *P* value noted<br>*Give P values as exact values whenever suitable.*
☒	☐	For Bayesian analysis, information on the choice of priors and Markov chain Monte Carlo settings
☒	☐	For hierarchical and complex designs, identification of the appropriate level for tests and full reporting of outcomes
☒	☐	Estimates of effect sizes (e.g. Cohen's *d*, Pearson's *r*), indicating how they were calculated

Our web collection on statistics for biologists contains articles on many of the points above.

Software and code

Policy information about availability of computer code

Data collection	In silico drug screening and molecular dynamics data were collected and analyzed with Schrodinger software suite (Releases 2016-2021, Schrodinger, LLC) and Pymol (Version 2.3, The PyMOL Molecular Graphics System, Schrodinger, LLC). NMR data were collected and processed with Topspin software (Version 2.1, 2016, Bruker). FACS data were collected using the BD FACSDIVA 8.
Data analysis	Data and statistical comparisons were performed by Graph pad Prism 9. NMR data analyzed with qMDD (mddnmr v2.0), NMRPIPE (v.20210129) and analyzed using Analysis CCPNMR (3.0). Structural and small molecule data were analyzed with modules PHASE, EPIC, LIGPREP, MAESTRO, DESMOND using Schrodinger software suite version 2016-2021. Western blot data analyzed with Image Studies 3.1. Imaging data analyzed using FIJI software. FACS data analyzed using FlowJo v 10. Sequencing results were demultiplexed and converted to FASTQ format using Illumina bcl2fastq software. The sequencing reads were adapter and quality trimmed with Trimmomatic and then aligned to the mouse genome (build mm10/GRCm38) using the splice-aware STAR aligner. The featureCounts program was utilized to generate counts for each gene based on how many aligned reads overlap its exons. These counts were then normalized and used to test for differential expression using negative binomial generalized linear models implemented by the DESeq2 1.26.0 package.

For manuscripts utilizing custom algorithms or software that are central to the research but not yet described in published literature, software must be made available to editors and reviewers. We strongly encourage code deposition in a community repository (e.g. GitHub). See the Nature Portfolio guidelines for submitting code & software for further information.

Data

Policy information about availability of data

All manuscripts must include a data availability statement. This statement should provide the following information, where applicable:

- Accession codes, unique identifiers, or web links for publicly available datasets
- A description of any restrictions on data availability
- For clinical datasets or third party data, please ensure that the statement adheres to our policy

The RNA-seq data generated in this study have been deposited in NCBI Gene Expression Omnibus (GEO) under the accession code GSE186924 [https://www.ncbi.nlm.nih.gov/geo/query/acc.cgi?acc=GSE186924]. The following publicly available data sets were used in the production of this manuscript: PDB ID: 6JFL doi:10.2210/pdb6JFL/pdb; 2J69 doi:10.2210/pdb2J69/pdb. 6JFL, 6JFK doi:10.2210/pdb6JFK/pdb, 5GOE doi:10.2210/pdb5GOE/pdb, 1T3J doi:10.2210/pdb1T3J/pdb. All the information generated and analyzed is included in the manuscript and all figures have associated raw data that is provided as an Excel worksheet organized by figures (Data Source file). Source data are provided with this paper.

Field-specific reporting

Please select the one below that is the best fit for your research. If you are not sure, read the appropriate sections before making your selection.

☒ Life sciences ☐ Behavioural & social sciences ☐ Ecological, evolutionary & environmental sciences

For a reference copy of the document with all sections, see nature.com/documents/nr-reporting-summary-flat.pdf

Life sciences study design

All studies must disclose on these points even when the disclosure is negative.

Sample size	Sample size were designed based on previous experience with assays on related projects (Garner et al. Nat. Chem. Bio. 2019 DOI: 10.1038/s41589-018-0223-0, Amgalan et al. Nat. Cancer 2020 DOI: 10.1038/s43013-020-0039-1) and variability of the response deviating from the mean as presented in the graphs and figure legends. Sample sizes and statistical data are reported in figure legends.
Data exclusions	No data were excluded from the analyses.
Replication	Technical replicates and independent experiments were performed to verify reproducibility of the assays. The experimental findings were reliably reproduced as described in the figure legends. Experimental findings were replicated at least 2 or more times as indicated in the figure legends and replication was successful at all attempts.
Randomization	Allocation was random. For biochemical and studies in cell culture, treatment groups were attributed randomly between wells and plates to account for well or plate positioning effects.
Blinding	Blinding was not required for this study as all comparisons were made using quantitative analysis of computational, biochemical, or cellular data with no human or animal subjects.

Reporting for specific materials, systems and methods

We require information from authors about some types of materials, experimental systems and methods used in many studies. Here, indicate whether each material, system or method listed is relevant to your study. If you are not sure if a list item applies to your research, read the appropriate section before selecting a response.

Materials & experimental systems

n/a	Involved in the study	
☐	☒	Antibodies
☐	☒	Eukaryotic cell lines
☒	☐	Palaeontology and archaeology
☒	☐	Animals and other organisms
☒	☐	Human research participants
☒	☐	Clinical data
☒	☐	Dual use research of concern

Methods

n/a	Involved in the study	
☒	☐	ChIP-seq
☐	☒	Flow cytometry
☒	☐	MRI-based neuroimaging

Antibodies

Antibodies used	β-Actin (clone AC-15): (Sigma; Cat. #1978) 1:5000 dilution<br>β-Tubulin (clone D3U1W): (Cell Signaling Technology; Cat. #86298) 1:1000 dilution<br>Phospho-Histone H2A.X (Ser139) (clone JBW301): (Sigma; Cat. #05-636) 1:100 dilution

	Tom20 (clone D8T4N): (Cell Signaling Technology; Cat. #42406) 1:1000 dilution<br>VDAC (clone D73D12): (Cell Signaling Technology; Cat. #4661) 1:1000 dilution<br>Cytochrome c (clone 7H8.2C12, BD): (Fisher Scientific; Cat. # BDB556433) 1:100<br>Mitofusin-2 (clone D2D10): (Cell Signaling Technology; Cat. #9482) 1:500 dilution<br>IRDye® 800CW Goat anti-Rabbit IgG Secondary Antibody: (LI-COR Biosciences; Cat. #926-32211) 1:5000 dilution<br>IRDye® 680RD Goat anti-Mouse IgG Secondary Antibody: (LI-COR Biosciences; Cat. #926-68070) 1:5000 dilution<br>Anti-mouse IgG HRP-linked Antibody: (Cell Signaling Technology; Cat. #7076) 1:2000 dilution<br>Anti-Rabbit IgG HRP-linked Antibody: (Sigma; Cat. #A4914) 1:2000 dilution
Validation	All antibodies used in this study were from commercial resources and have been validated in the literature and our previous previous publications. Such information is provided in the manufacturer's website.<br>β-Actin (clone AC-15), Sample PMID: 33602934<br>β-Tubulin (clone D3U1W), Sample PMID: 34136390<br>Phospho-Histone H2A.X (Ser139) (clone JBW301), Sample PMID: 20440269<br>Tom20 (clone D8T4N), Sample PMID: 35250566<br>VDAC (clone D73D12), Sample PMID: 35271667<br>Cytochrome c (clone 7H8.2C12, BD), Sample PMID: 29017059<br>Mitofusin-2 (clone D2D10), Sample PMID: 33637715<br>IRDye® 800CW Goat anti-Rabbit IgG Secondary Antibody, Sample PMID:31819006<br>IRDye® 680RD Goat anti-Mouse IgG Secondary Antibody, Sample PMID:31819006<br>Anti-mouse IgG HRP-linked Antibody, Sample PMID: 35121725<br>Anti-Rabbit IgG HRP-linked Antibody, Sample PMID: 24959379

Eukaryotic cell lines

Policy information about cell lines

Cell line source(s)	WT, MFN1 KO, MFN2 KO and MFN1/MFN2 DKO Mouse Embryonic Fibroblasts (referred to as WT, MFN1 KO, MFN2 KO, MFN1/MFN2 DKO MEFs) were provided by the laboratory of David Chan. U2OS were provided by the laboratory of Stephen Tait. APAF-1 KO MEFs were provided by Xuejun Jiang's laboratory. DRP1 KO MEFs were generated in house.
Authentication	Cell lines were authenticated from the providing lab. WT, MFN1 KO, MFN2 KO and MFN1/MFN2 DKO MEFs, APAF-1 KO MEFs, DRP1 KO MEFs were confirmed for lack of expression or expression of the corresponding proteins.
Mycoplasma contamination	All cell lines were tested negative for mycoplasma contamination.
Commonly misidentified lines (See ICLAC register)	No commonly misidentified lines were used in this study.

Flow Cytometry

Plots

Confirm that:

☒ The axis labels state the marker and fluorochrome used (e.g. CD4-FITC).

☒ The axis scales are clearly visible. Include numbers along axes only for bottom left plot of group (a 'group' is an analysis of identical markers).

☐ All plots are contour plots with outliers or pseudocolor plots.

☒ A numerical value for number of cells or percentage (with statistics) is provided.

Methodology

Sample preparation	Annexin V/PI staining was performed according to Dead Cell Apoptosis Kit manual (Invitrogen, V13241). Briefly, MEFs were detached from plates using accutase and centrifuged. Cells were resuspended in 100 µl of lX annexin-binding buffer containing Alexa Fluor 488 Annexin V and incubated for 15 minutes at room temperature in the dark. Subsequently, 400 µl of lX annexin-binding buffer were added. PI was added prior analysis with a LSRII Flow Cytometer.
Instrument	BD Bioscience LSRII Flow Cytometer
Software	For data analysis: FlowJo v 10 (Tree Star)<br>For data acquisition: BD FACSDiva 8 software
Cell population abundance	No cell sorting was performed.
Gating strategy	After cells were selected in the FSC/SSC dot plot to remove debris, they were gated to exclude cellular aggregates in the FSC/FSC and FSC/SSC dot plots. Gates of PE-Tx-Red-YG+ and FITC+ cells were set and compared with unstained and stained control samples.

☒ Tick this box to confirm that a figure exemplifying the gating strategy is provided in the Supplementary Information.
